# Development of a preoperative risk score for predicting blood transfusion in pediatric scoliosis surgery: a two-center retrospective cohort study

**DOI:** 10.3389/fmed.2026.1838600

**Published:** 2026-04-23

**Authors:** Peng Gao, Yuguan Zhang, Zhengzheng Gao, Jianmin Zhang, Jinhua Bo, Xiaoping Gu, Li Xu, Bo Zhu, Yuguang Huang

**Affiliations:** 1 Department of Anesthesiology, Peking Union Medical College Hospital, Chinese Academy of Medical Sciences and Peking Union Medical College, Beijing, China; 2 Department of Anesthesiology, Beijing Children’s Hospital, Capital Medical University, National Center for Children’s Health, Beijing, China; 3 Department of Anesthesiology, Drum Tower Clinical College, Nanjing Medical University, Nanjing, China

**Keywords:** blood transfusion, pediatrics, prediction model, risk score, scoliosis surgery

## Abstract

**Background:**

Despite recent advancements in blood conservation strategies, perioperative allogeneic red blood cell (RBC) transfusion remains common in pediatric patients undergoing scoliosis surgery. This study aimed to develop a simplified preoperative risk score to predict allogeneic RBC transfusion requirements in this population.

**Methods:**

We conducted a retrospective cohort study of 1,992 pediatric patients (<18 years) who underwent scoliosis surgery at two tertiary care centers between January 2018 and May 2023. The primary outcome was perioperative allogeneic RBC transfusion. Missing data were addressed via multiple imputation, generating 10 imputed datasets. Predictor variables were screened through univariate and least absolute shrinkage and selection operator (LASSO) regression. Multivariable logistic regression was used to develop the prediction model, followed by stepwise variable selection to identify a parsimonious set of predictors without compromising model performance. A simplified risk score and corresponding risk stratification system were derived from the final model coefficients.

**Results:**

The overall perioperative RBC transfusion rate was 32.9% (655/1,992). After variable selection and model optimization, the final prediction model included four preoperative variables: American Society of Anesthesiologists (ASA) physical status grade, Cobb angle, diagnosis, and weight. The resulting risk score ranged from 0 to 20 points and exhibited strong discriminative ability, with an area under the receiver operating characteristic curve (AUC) of 0.818 [95% confidence interval (CI): 0.793–0.843] in the training set and 0.814 (95% CI: 0.764–0.864) in the validation set. Three risk categories were established: low risk (0–4 points), medium risk (5–10 points), and high risk (≥11 points). Transfusion rates exhibited a progressive increase across risk categories in both training (9.6, 37.3, 76.3%) and validation (7.3, 32.1, 74.1%) sets.

**Conclusion:**

We developed a simplified risk score based on four readily available preoperative variables (diagnosis, weight, Cobb angle, and ASA grade) to predict perioperative allogeneic RBC transfusion in pediatric scoliosis surgery. This tool facilitates preoperative transfusion risk assessment and stratification, serving as a practical clinical aid that may contribute to optimized perioperative blood management strategies.

## Introduction

Scoliosis most commonly presents in children during periods of rapid growth, with severe cases often requiring surgical intervention (1). Posterior spinal fusion (PSF), the standard procedure for severe scoliosis, involves steps such as screw placement and corticotomies (2). Given the substantial surgical trauma and prolonged operative duration associated with this procedure, significant intraoperative blood loss is a common occurrence (3). While surgical techniques have advanced markedly in recent years (4–6), and blood conservation strategies (e.g., antifibrinolytic agents, intraoperative autologous blood salvage) have been widely implemented (7, 8), perioperative allogeneic red blood cell (RBC) transfusion rates in pediatric scoliosis surgery remain persistently high, ranging from approximately 20 to 50% across different institutions and patient cohorts (9–11).

Perioperative allogeneic RBC transfusion is generally linked to adverse outcomes, including transfusion reactions and healthcare-associated infections (12); in the context of pediatric scoliosis surgery, transfusion has also been associated with poorer clinical prognosis (13–15). Although numerous risk factors for transfusion in this population have been identified in prior studies (16), existing predictive tools are often limited in clinical practice. For example, Eisler et al. (17) developed a 10-item transfusion risk score using the ACS NSQIP-P database, but its utility is hampered by the complexity of collecting and calculating scores from ten distinct variables. In contrast, a simplified risk score that incorporates fewer, preoperatively accessible variables would enable more efficient risk assessment and better support clinical decision-making.

Therefore, the primary objective of the present study was to develop a simplified preoperative risk score to predict perioperative allogeneic RBC transfusion in pediatric patients undergoing scoliosis surgery. We hypothesize that this tool will enhance preoperative risk stratification and facilitate the implementation of individualized perioperative blood management strategies.

## Methods

### Study design and population

This retrospective cohort study enrolled pediatric patients (aged <18 years) who underwent scoliosis surgery at two major spinal deformity treatment centers in China—Peking Union Medical College Hospital (Center 1) and Beijing Children’s Hospital Affiliated to Capital Medical University (Center 2)—between January 2018 and May 2023. Exclusion criteria included emergency surgeries, reoperations or revisions for scoliosis, American Society of Anesthesiologists (ASA) physical status grade IV, and cases with >30% missing perioperative clinical data.

Data were automatically extracted from the electronic medical record systems of both institutions and subsequently subjected to manual collation and verification. To ensure data accuracy, extreme outliers (e.g., laboratory values outside clinically plausible ranges) were cross-referenced with original medical records. Additionally, two patients who had received preoperative blood transfusions were excluded from the analysis. This exclusion was justified by the exceptionally low prevalence (0.1%) and the fact that the transfusions were attributable to underlying comorbidities rather than perioperative surgical factors.

The study protocol was approved by the institutional review boards (IRBs) of both participating centers prior to data acquisition. Given the retrospective nature of the research and the use of de-identified patient data, the requirement for informed consent was waived by the respective IRBs. This study was conducted and reported in accordance with the Transparent Reporting of a Multivariable Prediction Model for Individual Prognosis or Diagnosis (TRIPOD) guidelines (18) as well as the Strengthening the Reporting Of Cohort Studies in Surgery (STROCSS) guidelines (19).

### Clinical practice

All patients underwent posterior spinal fusion (PSF) performed by experienced senior surgeons (≥10 years of clinical experience in scoliosis surgery). A midline posterior approach was used, with detachment of paravertebral muscles via electrocautery. Pedicle screws were inserted using the free-hand technique, and hemostasis was achieved with bipolar electrocoagulation and bone wax. A subfascial drain was routinely placed at the conclusion of the procedure and removed 48–72 h postoperatively.

All patients received general anesthesia with endotracheal intubation, induced and maintained primarily with propofol and fentanyl. Following anesthesia induction, invasive blood pressure was monitored via radial artery cannulation as directed by senior anesthesiologists. A loading dose of tranexamic acid (1 g) and prophylactic antibiotics were administered prior to skin incision. Autologous blood salvage was utilized in all cases, and intermittent arterial blood gas analysis was performed to guide transfusion decisions. Allogeneic RBC transfusion was initiated if hemoglobin (Hb) levels fell below 70 g/L; for Hb levels between 70 and 100 g/L, transfusion was administered if clinical signs of hypovolemia (e.g., hemodynamic instability) were present. These transfusion criteria were consistently applied during both the intraoperative and postoperative periods.

The primary outcome was defined as the administration of allogeneic RBC transfusion either intraoperatively or within the first 7 postoperative days. Autologous blood salvaged intraoperatively and other allogeneic blood products (e.g., fresh frozen plasma, platelets) were excluded from this outcome. Transfusion events were independently assessed by two researchers based on electronic medical records; any discrepancies were resolved through consensus following re-examination of the original clinical documentation.

### Statistical analysis

#### Data partitioning and missing data imputation

The entire cohort was randomly divided into a training set and a validation set in a 7:3 ratio using stratified random sampling based on the transfusion outcome. This sampling method was adopted to ensure balanced distribution of transfusion events between the two sets, thereby reducing sampling bias and enhancing the reliability of model validation. Baseline characteristics of both sets were summarized as follows: continuous variables were presented as median [interquartile range (IQR)], and categorical variables as count (percentage). Group comparisons were performed using the Chi-square test for categorical variables and the Mann–Whitney *U* test for continuous variables, as appropriate.

Missing data in the training set were addressed via multiple imputation using the mice package in R, with predictive mean matching (PMM) to generate 10 imputed datasets (20 iterations each). Parameter estimates and 95% confidence intervals (CI) from these datasets were pooled using Rubin’s rules via the pool() function. For the validation set, a complete-case analysis was adopted, with patients excluded if they had any missing covariate data, resulting in a final validation dataset for internal validation.

#### Model development and optimization

Univariate logistic regression was performed on each of the 10 imputed training datasets to evaluate associations between candidate predictors and the transfusion outcome. Feature selection was conducted using least absolute shrinkage and selection operator (LASSO) regression with 10-fold cross-validation, and the optimal penalty parameter (lambda) was selected using the ‘lambda.1se’ criterion. Variables retained at this lambda value were carried forward for further modeling, and two multivariable logistic regression models were developed:

*Model 1:* Included variables with *p* < 0.1 from univariate analysis, refined via bidirectional stepwise selection based on the Akaike Information Criterion (AIC) using the stepAIC function.

*Model 2:* Incorporated variables identified by LASSO regression at lambda.1se.

Both models were fitted across all 10 imputed datasets, with coefficients pooled using Rubin’s rules. Model performance was assessed using the area under the receiver operating characteristic curve (AUC) for discrimination and the Hosmer–Lemeshow (HL) test for calibration (*p* > 0.05 indicated adequate calibration). Given comparable performance between models, the more parsimonious Model 2 was selected for further refinement. Bidirectional stepwise regression (stepAIC) was then applied across all imputed datasets to exclude variables that were infrequently selected or had limited clinical relevance. The resulting reduced model (designated *Model 3*) was refitted and evaluated based on AUC, calibration, sensitivity, and specificity.

#### Development of the transfusion risk score

Continuous variables in the final model were categorized to enhance clinical applicability. Restricted cubic splines (RCS) were used to assess the functional form of continuous predictors and identify appropriate cutoff points; in the absence of nonlinearity, variables were categorized based on quartiles. The model was then refitted using these categorized variables.

A points-based risk score was developed by converting pooled regression coefficients from the grouped logistic model to integer weights, scaled relative to the smallest absolute coefficient. The total score for each patient was calculated as the sum of individual item scores. Risk stratification was performed by dividing total scores into three clinically meaningful tiers based on observed transfusion probabilities. The discriminative ability of the simplified scoring system was evaluated by AUC, and calibration was assessed by grouping patients into deciles of predicted risk and comparing mean predicted probabilities with observed event rates within each decile. Transfusion rates for each risk stratum were computed across all 10 imputed datasets to ensure robustness.

All analyses were conducted using R software (version 4.5.1) with the following packages: tidyverse, glmnet, pROC, rms, and caret.

## Results

### Study population and dataset partitioning

**Figure 1:**
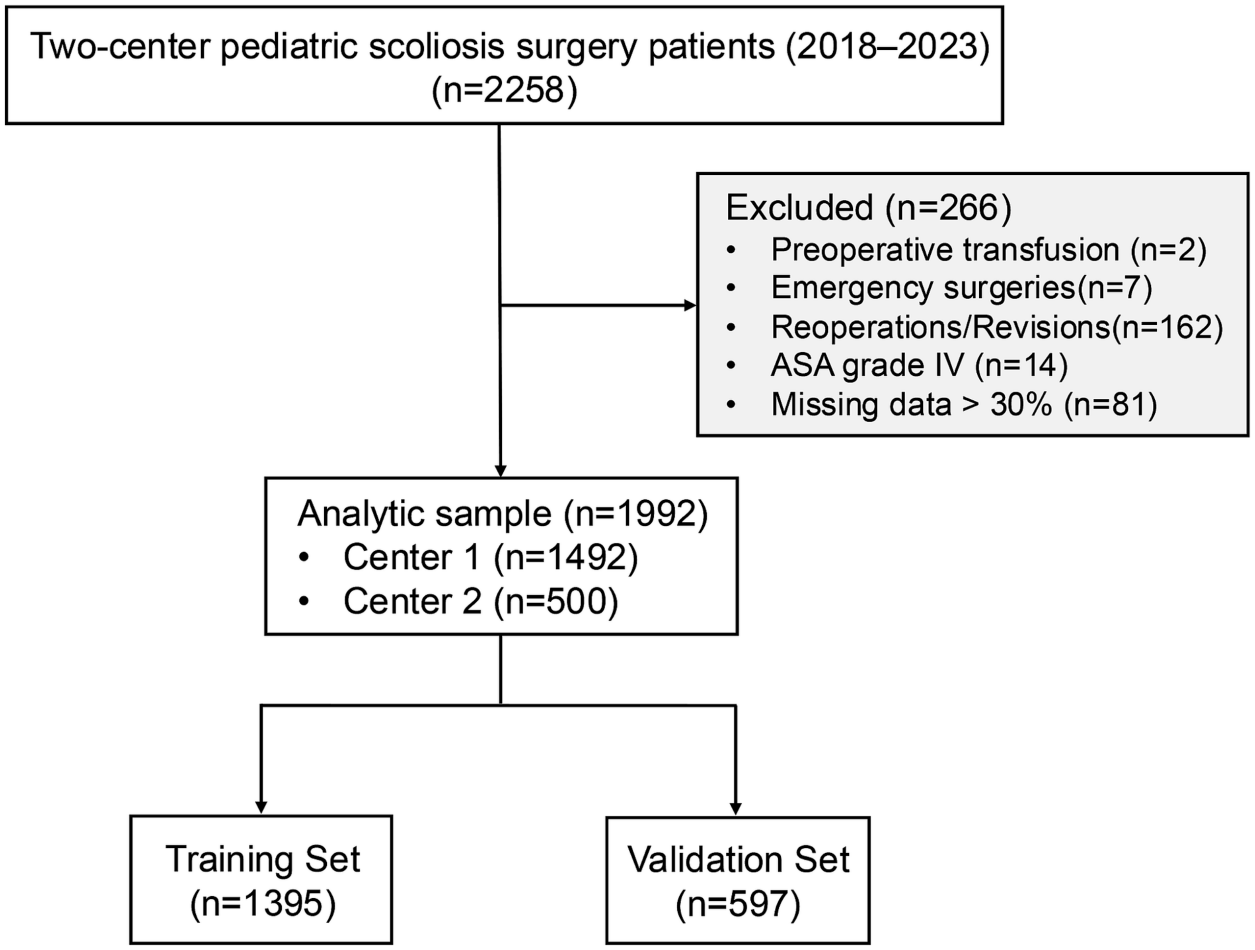
Study flowchart. ASA, American Society of Anesthesiologists.

As shown in Figure 1, a total of 2,258 pediatric patients who underwent scoliosis surgery at the two participating centers between January 2018 and May 2023 were initially screened. Of these, 266 patients were excluded based on the following criteria: reoperations or revisions for scoliosis (*n* = 162), emergency procedures (*n* = 7), American Society of Anesthesiologists (ASA) physical status grade IV (*n* = 14), preoperative blood transfusion (*n* = 2), and missing >30% of perioperative clinical data (*n* = 81). The final analytic cohort included 1992 patients, with 1,492 from Center 1 and 500 from Center 2.

The study population had a mean (± standard deviation) age of 11.4 ± 4.1 years, weight of 40.8 ± 17.0 kg, and height of 146 ± 24.5 cm. Males accounted for 34.1% of the cohort, and 7.4% (*n* = 148) were classified as ASA grade III. Diagnostic subtypes of scoliosis included idiopathic (52.7%, *n* = 1,049), congenital (33.1%, *n* = 660), neuromuscular (4.6%, *n* = 92), neurofibromatosis (4.8%, *n* = 96), and syndromic scoliosis (4.8%, *n* = 95). The overall perioperative allogeneic RBC transfusion rate was 32.9%, with center-specific rates of 36.3% (Center 1) and 22.8% (Center 2).

The entire cohort was randomly divided into a training set (*n* = 1,395) and a validation set (*n* = 597) in a 7:3 ratio via stratified random sampling. Comparisons of baseline characteristics between the two sets revealed no statistically significant differences across any variables (all *p* > 0.05; Table 1), confirming that the training and validation sets were well-balanced at baseline.

**Table 1:** Baseline characteristics of pediatric patients with scoliosis.

Variable	Training set	Validation set	*p* value
	*n* = 1,395	*n* = 597	
Demographics			
Age (year)	13 [10, 14]	13 [9, 14]	0.975
Gender (male)	485 (34.8%)	195 (32.7%)	0.392
Height (cm)	155 [132.5, 164]	155 [129, 164]	0.427
Weight (kg)	42.6 [29, 51]	42.0 [26, 50]	0.252
Clinical features			
Center 1 (%)	1,058 (75.8%)	434 (72.7%)	0.154
ASA grade III	100 (7.2%)	48 (8.0%)	0.558
Cobb angle (°)	45 [34, 56.5]	43 [32, 55]	0.363
RBC transfusion	459 (32.9%)	196 (32.8%)	1.000
Surgical duration (min)	224 [181, 278]	227 [175, 283]	0.832
Diagnosis (%)			0.552
Idiopathic	729 (52.3%)	320 (53.6%)	
Congenital	457 (32.8%)	203 (34%)	
Neurofibromatosis	71 (5.1%)	21 (3.5%)	
Neuromuscular	69 (4.9%)	27 (4.5%)	
Syndromic	69 (4.9%)	26 (4.4%)	
Laboratory parameters			
Hemoglobin (g/L)	133 [126, 142]	133 [125.5, 141]	0.474
Hematocrit (%)	39.7 [37.5, 42]	39.5 [37.5, 41.6]	0.526
Platelet count (10^9^/L)	264 [226, 311]	268 [229, 314.5]	0.510
White blood cell (10^9^/L)	6.3 [5.4, 7.6]	6.3 [5.4, 7.5]	0.812
Albumin (g/L)	44 [42, 46]	43.9 [42, 45.7]	0.108
ALT (U/L)	11 [9, 15]	11 [9, 14.9]	0.202
AST (U/L)	20 [17, 26.9]	20 [17, 27]	0.848
Total bilirubin (μmol/L)	9.9 [7.5, 13.2]	9.7 [7.2, 13.1]	0.237
Direct bilirubin (μmol/L)	2.5 [1.7, 3.7]	2.3 [1.6, 3.7]	0.112
Serum creatinine (μmol/L)	46 [36, 55]	47 [36, 56]	0.819
BUN (μmol/L)	4.3 [3.7, 5]	4.3 [3.6, 5.1]	0.883
Fibrinogen (g/L)	2.3 [2, 2.7]	2.3 [2, 2.6]	0.584
Prothrombin time (s)	12.1 [11.6, 12.7]	12.1 [11.6, 12.6]	0.349
APTT (s)	30.2 [28.2, 33.3]	30.4 [28.1, 33.4]	0.724
INR	1 [1, 1.1]	1 [1, 1.1]	0.954

Data are presented as median [interquartile range] for continuous variables and counts (percentages) for categorical variables. Comparisons between groups were performed using the Mann–Whitney *U* test and Chi-square test. ASA, American Society of Anesthesiologists; RBC, red blood cell; ALT, alanine aminotransferase; AST, aspartate aminotransferase; BUN, blood urea nitrogen; APTT, activated partial thromboplastin time; INR, international normalized ratio.

### Missing data handling

A substantial proportion of missing data was observed, with the highest rate for Cobb angle (29.5% in the training set and 27.8% in the validation set). Under the assumption of missingness at random (MAR), multiple imputation was performed on the training set to generate 10 imputed datasets. Subsequent analyses were conducted on each imputed dataset, and results were pooled using Rubin’s rules. For the validation set, a complete-case analysis was adopted: all cases with any missing covariate data were excluded, resulting in a final validation set of 356 patients for internal validation.

### Model development and comparison

Univariate logistic regression was performed on the 10 imputed training datasets to evaluate associations between candidate variables and the transfusion outcome (Table 2). Subsequently, LASSO regression was applied to each imputed dataset for variable selection. The mean number of variables selected was 20.7 ± 1.49 at the minimum lambda value and 7.4 ± 0.97 at lambda.1se. To optimize model parsimony, variables consistently retained at lambda.1se with a selection frequency exceeding 80% were included for further analysis. These variables were ASA grade, Cobb angle, diagnostic subtype, height, weight, age, and activated partial thromboplastin time (APTT).

**Table 2:** Univariate logistic regression analysis for RBC transfusion in the training set.

Variable	OR	95%CI	*p* value
ASA grade III	7.54	4.69–12.12	<0.001
Age (year)	0.89	0.86–0.91	<0.001
Cobb angle (°)	1.04	1.03–1.05	<0.001
Congenital scoliosis	6.26	4.76–8.22	<0.001
Neuromuscular scoliosis	12.27	7.14–21.09	<0.001
Height (cm)	0.98	0.97–0.98	<0.001
Weight (kg)	0.97	0.96–0.97	<0.001
Serum creatinine (μmol/L)	0.96	0.95–0.97	<0.001
Total bilirubin (μmol/L)	0.93	0.91–0.96	<0.001
Syndromic scoliosis	3.99	2.38–6.71	<0.001
AST (U/L)	1.04	1.02–1.05	<0.001
Neurofibromatosis scoliosis	3.33	1.97–5.64	<0.001
APTT (s)	0.94	0.91–0.97	<0.001
Platelet count (10^9^/L)	1.003	1.002–1.005	<0.001
Hemoglobin (g/L)	0.98	0.97–0.99	<0.001
Hematocrit (%)	0.95	0.91–0.98	<0.001
Gender (male)	1.43	1.14–1.8	0.002
Direct bilirubin (μmol/L)	0.92	0.86–0.98	0.010
INR	0.30	0.06–1.58	0.150
Blood urea nitrogen (μmol/L)	1.08	0.97–1.21	0.170
Albumin (g/L)	0.97	0.94–1.02	0.220
Fibrinogen (g/L)	0.90	0.72–1.12	0.340
White blood cell (10^9^/L)	1.02	0.96–1.09	0.460
ALT (U/L)	1.00	0.99–1.01	0.950
Prothrombin time (s)	1.00	0.86–1.16	0.990

Results were pooled across 10 imputed datasets using Rubin’s rules. RBC, red blood cell; OR, odds ratio; CI, confidence interval; ASA, American Society of Anesthesiologists; AST, aspartate aminotransferase; APTT, activated partial thromboplastin time; INR, international normalized ratio; ALT, alanine aminotransferase.

Variables with a univariate association with perioperative allogeneic RBC transfusion (*p* < 0.1) were incorporated into a multivariable logistic regression model (*Model 1*), which was then refined via bidirectional stepwise selection based on the Akaike Information Criterion (AIC) using the stepAIC function. In parallel, a separate multivariable logistic regression model (*Model 2*) was developed using the variables selected by LASSO regression at lambda.1se.

*Model 1* retained 14 variables, while *Model 2* included 7 variables. For discriminative ability, *Model 1* exhibited a marginally higher mean AUC of 0.830 (95%CI: 0.806–0.854) compared to 0.825 (95%CI: 0.801–0.849) for *Model 2*, with comparable standard deviations (0.004). These findings indicate negligible differences in discriminatory power and similar model stability in the training set. Both models demonstrated good calibration, as evidenced by Hosmer–Lemeshow (HL) test results (*Model 1*: *p* = 0.401; Model 2: *p* = 0.573), with *Model 2* showing slightly better calibration. Sensitivity and specificity were also comparable between models (Supplementary Table 1), confirming similar clinical discriminative ability. Model performance remained consistent in the validation set (AUC: 0.813 vs. 0.811). Given its superior parsimony and non-inferior predictive performance, *Model 2* was selected for further simplification to develop a clinically applicable transfusion risk score.

### Model simplification

To enhance clinical applicability, bidirectional stepwise regression was applied to *Model 2* for further variable selection. This procedure was performed separately on each of the 10 imputed datasets, with variables iteratively included or excluded to minimize the AIC. The stability of each variable was evaluated based on its frequency of selection across all imputed datasets, with higher selection frequency indicating a more robust association with transfusion risk and a greater contribution to model fit.

The results indicated that “Height” was not selected in any imputed dataset, while “Age” was retained in only 50% of datasets. Consequently, both variables were excluded from the final model. Although APTT was frequently selected, it was associated with a modest effect size [odds ratio (OR) = 0.938, 95% confidence interval (CI): 0.901–0.975]. Removal of APTT did not substantially compromise model performance, as evidenced by an AUC of 0.821 (95%CI 0.797–0.846) in the training set and 0.813 in the validation set—values comparable to those of the original *Model 2* (Supplementary Table 1). Although APTT was frequently selected, its modest effect size and limited incremental predictive value, together with a goal of maximizing clinical practicality and minimizing required preoperative variables, supported its exclusion from the final score. Thus, a simplified four-variable model (*Model 3*) was finalized for subsequent risk score development. The regression coefficients of the retained variables are presented in Table 3.

**Table 3:** Variables and statistical characteristics of the final four-variable model (*Model 3*).

Variable	OR	95% CI	*p* value
Neurofibromatosis scoliosis	1.74	0.931–3.27	0.082
Congenital scoliosis	4.69	3.45–6.38	<0.001
Neuromuscular scoliosis	6.77	3.62–12.7	<0.001
Syndromic scoliosis	3.12	1.67–5.81	<0.001
ASA grade III	4.82	2.80–8.31	<0.001
Cobb angle (°)	1.03	1.02–1.04	<0.001
Weight (kg)	0.968	0.960–0.977	<0.001

OR, odds ratio; CI, confidence interval; ASA, American Society of Anesthesiologists.

### Risk score development and risk stratification

To enhance clinical applicability, all variables in *Model 3* were categorized. For continuous variables (Cobb angle and weight), RCS were used to evaluate their functional relationship with transfusion outcomes. The analysis revealed no significant inflection points, indicating an approximately linear association for both variables. To maximize clinical utility, these variables were categorized into three groups based on integer quartile-derived thresholds: weight was classified as <30 kg, 30–50 kg, and >50 kg (median: 42.6 kg; IQR: 29–51 kg); Cobb angle was classified as <35°, 35–55°, and >55° (median: 45°; IQR: 34–56.5°).

**Figure 2:**
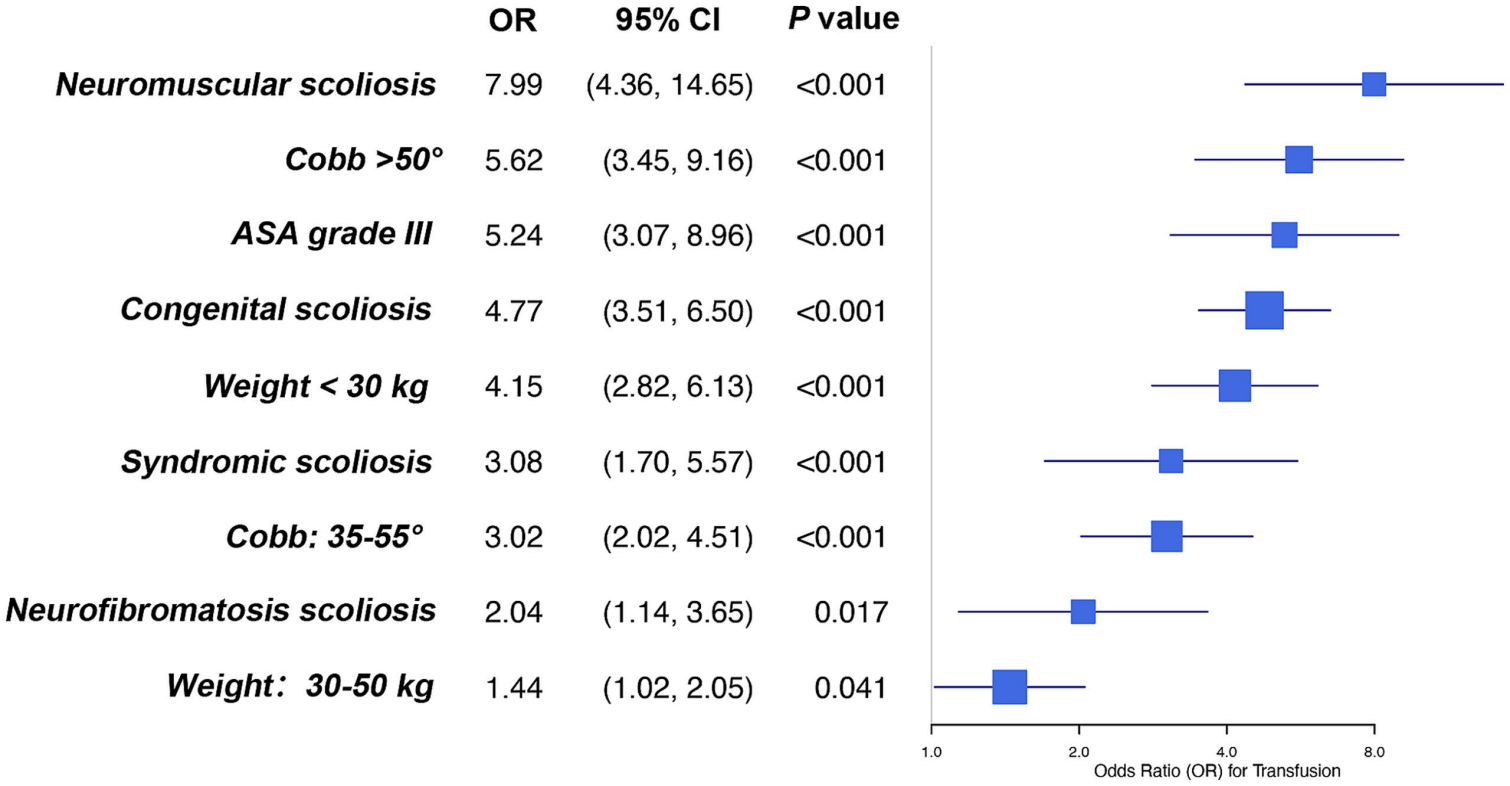
Coefficients of the final risk score model with categorized variables. ASA, American Society of Anesthesiologist; OR, odds ratio; CI, confidence interval.

**Figure 3:**
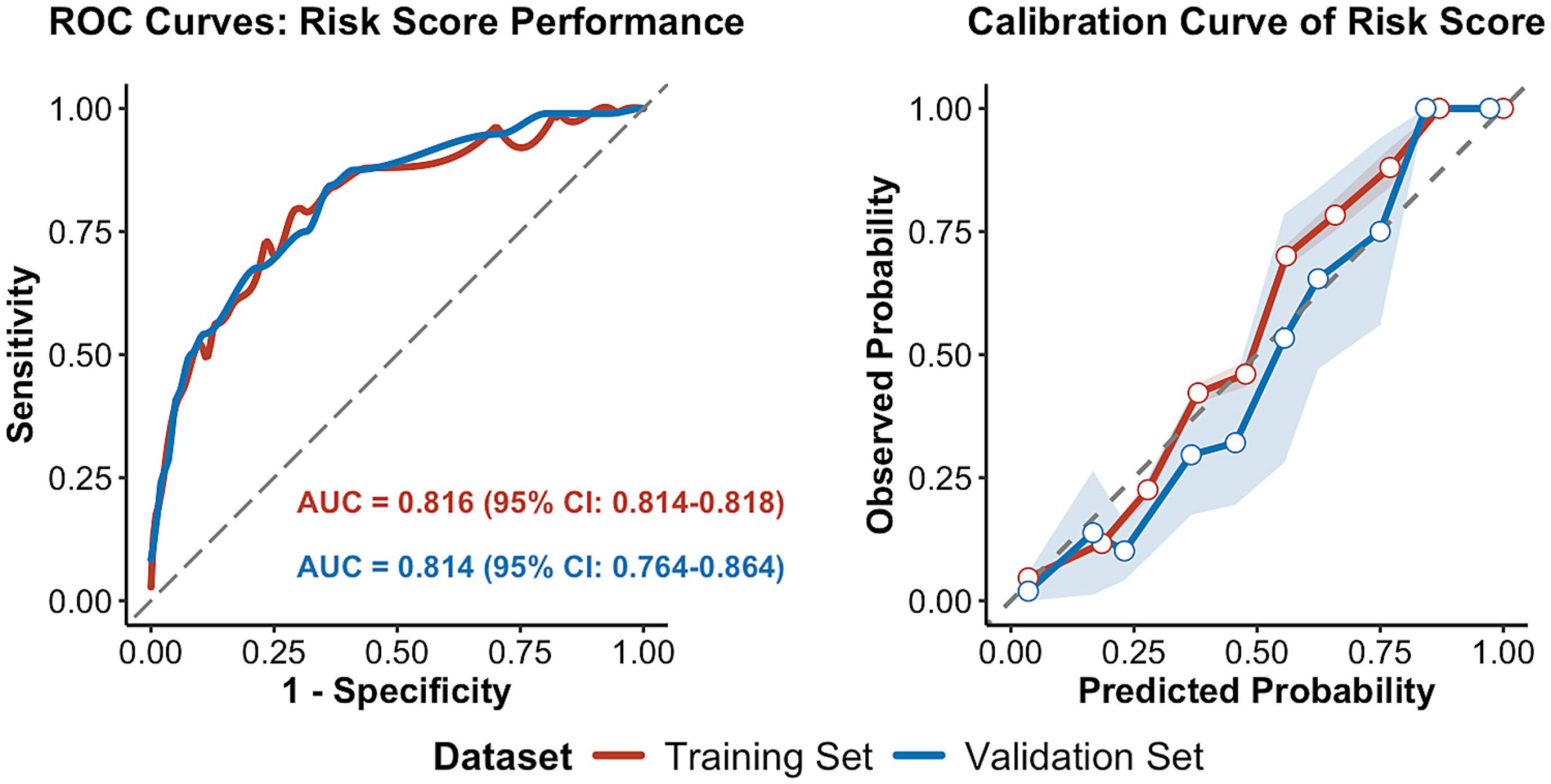
Risk score model: ROC and calibration curves in training and validation sets. Validation set results are based on complete-case analysis (*n* = 356). ROC, receiver operating characteristic; AUC, area under the ROC curve.

A risk prediction model was refitted using these categorized variables, with corresponding regression coefficients presented in Figure 2. The model exhibited strong discriminative ability, with an AUC of 0.818 (95% CI: 0.793–0.843) in the training set and 0.814 (95% CI: 0.764–0.864) in the complete-case validation set (*n* = 356) (Figure 3). Model calibration was satisfactory, as evidenced by HL test *p*-values of 0.287 (training set) and 0.083 (validation set). Calibration curves further confirmed close agreement between predicted and observed transfusion probabilities in both datasets (Figure 3).

**Figure 4:**
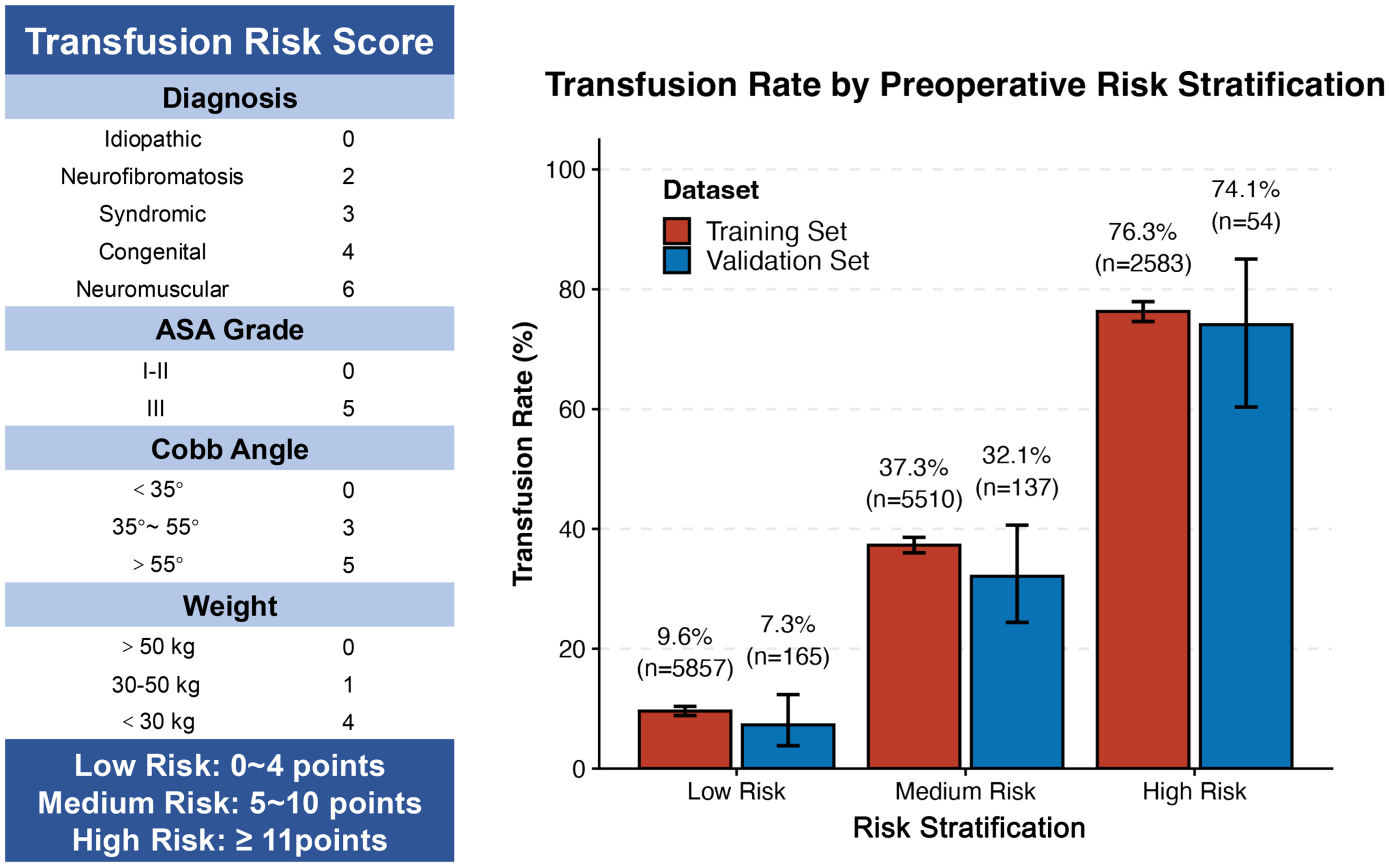
Transfusion risk score: scoring rules and stratified transfusion rates. *Left panel*: Risk scoring rules. The transfusion risk score is based on four predictors, with each category assigned a specific score. The total risk score (range: 0–20 points) is the sum of individual item scores. *Example*: A patient with ASA grade II (0 points), Cobb angle 40° (3 points), congenital scoliosis (4 points), and weight 40 kg (1 point) has a total score of 8. *Right panel*: Transfusion rates by risk stratification. Bar plots show observed transfusion rates (with 95% CI) for each risk stratum. Training set results are pooled across 10 imputed datasets; validation set results are based on a complete-case analysis.

Scoring rules were then derived from the model coefficients (Figure 4), with total scores ranging from 0 (lowest risk) to 20 (highest risk). Based on score-specific transfusion rates observed in the pooled training set (Supplementary Table 2), three distinct risk categories were established: low risk (0–4 points), medium risk (5–10 points), and high risk (≥11 points). Transfusion rates differed significantly across these strata in both training and validation sets (Figure 4), supporting the clinical utility of the stratification system.

In the pooled training set, transfusion rates were 9.6% (95% CI: 8.9–10.4) in the low-risk group, 37.3% (95% CI: 36.1–38.5) in the medium-risk group, and 76.3% (95% CI: 74.7–77.8) in the high-risk group. Corresponding rates in the validation set were 7.3% (95% CI: 3.9–12.2), 32.1% (95% CI: 24.3–40.7), and 74.1% (95% CI: 60.7–84.7), respectively. Although minor numerical variations were observed—consistent with expected differences in model validation—a strong, consistent upward trend in transfusion rates was evident across increasing risk categories, further supporting the validity of the risk score.

## Discussion

In the present study, we developed a simplified preoperative risk score to predict perioperative allogeneic RBC transfusion in pediatric patients undergoing scoliosis surgery. The final model incorporated four readily accessible preoperative variables: diagnosis, weight, Cobb angle, and ASA grade, and exhibits good discriminative ability, with AUC of 0.818 (95% CI: 0.793–0.843) in the training and 0.814 (95% CI: 0.764–0.864) in the validation set. Notably, transfusion rates showed a consistent upward gradient across the three derived risk strata in both cohorts: in the training set, rates were 9.6% (low risk, 0–4 points), 37.3% (medium risk, 5–10 points), and 76.3% (high risk, ≥11 points), with corresponding rates of 7.3, 32.1, and 74.1% in the validation set. This distinct gradient confirms the score’s clinical utility for preoperative risk stratification and supports its use in guiding individualized perioperative blood management strategies.

Pediatric scoliosis surgery—specifically PSF—is characterized by complex procedures and substantial surgical trauma, often resulting in significant intraoperative bleeding and increased transfusion requirements (3). Perioperative allogeneic RBC transfusion rates vary widely across studies, with this heterogeneity attributed to differences in patient demographics, scoliosis diagnostic subtypes (20, 21), and institutional expertise or surgical experience (10). For example, Dick et al. from Imperial College London reported a transfusion rate of 24.4% following a 15-year quality improvement project (9), while O’Malley et al. (10) observed an overall rate of 21.1% in an analysis of 7,689 patients, highlighting substantial variability in transfusion practices. In contrast, Fercho et al. (22) reported a higher rate of 40.75% using national data from Poland.

Historical data from our institution (Center 1) also reflect substantial variability in transfusion-related outcomes, with a massive bleeding rate of 59.7% in 2011 (23) declining to a transfusion rate of 32.8% in a 2014–2017 cohort (24). In the present multicenter analysis, the overall transfusion rate was a comparable 32.9%. A center-specific breakdown, however, revealed a pronounced disparity: the rate remained higher at our institution (Center 1) at 36.3%, while it was notably lower at Center 2 (22.8%). This finding provides a clear example of the institutional heterogeneity in transfusion outcomes.

Multiple factors are associated with perioperative allogeneic RBC transfusion in pediatric scoliosis surgery, which can be broadly categorized into four domains: (1) Patient-specific factors, such as gender, age, weight, ASA grade, and preoperative comorbidities including anemia and cardiovascular disease (17, 25); (2) Scoliosis-related factors, including diagnostic subtype (e.g., idiopathic, congenital) and curve magnitude as quantified by the Cobb angle (26, 27); (3) Surgical factors, encompassing operative duration, surgical experience, number of fused vertebrae, and osteotomy techniques (e.g., Ponte osteotomy, hemivertebra resection) (10, 16); and (4) Anesthetic and perioperative management factors, such as fluid therapy, hemodynamic stability, temperature maintenance, and coagulation monitoring (13, 28).

Numerous studies have attempted to develop predictive tools for transfusion requirements in pediatric scoliosis surgery using these risk factors (16). In 2022, Eisler et al. (17) developed a transfusion risk score via analysis of the ACS NSQIP-P database, incorporating ten variables: race, gender, age, ASA grade, preoperative anemia, hematologic disorders, cardiac risk factors, weight, neuromuscular disease, and fusion levels. However, the need for ten variables complicates both data collection and score calculation, and the score’s generalizability may be limited by regional or healthcare system differences. To enhance clinical applicability and address these limitations, we aimed to retain the minimal number of predictive variables without substantially compromising model performance. Notably, the Cobb angle serves as a reliable preoperative surrogate for fusion levels (17), meaning all variables in our simplified score are encompassed within Eisler’s model. Furthermore, our 4-variable score features straightforward data collection, making it more suitable for rapid clinical assessment of transfusion risk while maintaining comparable or even slightly superior predictive performance [AUC: 0.818 vs. 0.77 reported by Eisler et al. (17)].

The four variables included in our risk score are routinely accessible preoperatively and collectively capture the key determinants of perioperative allogeneic RBC transfusion risk. Diagnostic subtype and Cobb angle reflect scoliosis severity, while ASA grade and weight reflect the patient’s overall physical status—supporting the clinical rationale for selecting these four variables to predict transfusion requirements (29). Specifically, neuromuscular scoliosis showed the strongest association with transfusion needs, consistent with earlier findings (17). Other studies have also identified diagnostic subtype as a key determinant of transfusion risk (26, 30), likely due to differences in inherent disease complexity and associated surgical challenge across subtypes (21). The Cobb angle is a well-established indicator of scoliosis severity, correlating with the extent of surgical trauma and intraoperative blood loss (25, 27), while ASA grade, widely used to assess preoperative health status, is positively associated with transfusion risk, as higher grades indicate more comorbidities and poorer baseline condition (31). Finally, patient weight is closely linked to total blood volume and nutritional status in pediatric patients; lower weight increases the likelihood of transfusion for a given volume of blood loss (25, 29).

While previous risk scores have emphasized the importance of anemia (17), forced inclusion of hemoglobin (Hb) in our final model did not improve performance, suggesting potential overfitting. This finding is consistent with another study indicating that preoperative RBC counts poorly predict intraoperative transfusion needs (32).

For pediatric scoliosis surgery, our simplified risk score offers a practical, readily applicable preoperative assessment tool with multiple potential clinical applications: (1) enabling anesthesiologists to implement more proactive blood conservation strategies (e.g., acute normovolemic hemodilution) for high-risk patients (33); (2) guiding surgical blood ordering practices to optimize resource utilization efficiency; (3) facilitating communication with guardians regarding transfusion risks to improve informed consent; and (4) identifying candidates for preoperative autologous blood preservation or iron supplementation therapy—particularly in cases involving rare blood types or transfusion refusal (34, 35).

Several limitations of this study should be acknowledged. First, the data were retrospectively extracted from electronic medical records. Although outliers were manually verified, a substantial proportion of missing values—particularly for the non-structured variable of Cobb angle—may affect the model’s generalizability. To address this, we handled data under the assumption of missingness at random and performed multiple imputation. Second, a major limitation is the lack of external validation. Despite the two-center design enhancing internal validity, the model’s generalizability across different medical centers remains to be confirmed. Third, we used conventional logistic regression to develop a transparent and clinically applicable score. While we acknowledge that its predictive performance may be inferior to more complex machine learning approaches, sensitivity analyses showed no significant improvement in discrimination when using either complete-case data or machine learning models (e.g., XGBoost). This is likely because perioperative transfusion is influenced by multiple intraoperative factors that are difficult to anticipate preoperatively. Additionally, the “black-box” nature of most machine learning models prevents the extraction of variable coefficients, making conversion into a simple, points-based risk score impractical. Finally, despite the two-center design, the sample size remains relatively limited, which constrains the precision of estimated transfusion rates for individual score values and may affect the stability of the risk categories.

## Conclusion

In conclusion, through a multi-step process of variable selection and model optimization, we developed a simplified preoperative risk score comprising four routinely available preoperative variables—diagnosis, weight, Cobb angle, and ASA grade—to predict perioperative allogeneic RBC transfusion in pediatric scoliosis surgery. This tool exhibits strong predictive performance and offers simplicity for clinical application, facilitating preoperative risk stratification and individualized blood management. Future research should focus on external validation of this score across diverse medical centers to enhance its generalizability, as well as developing risk stratification-specific interventions and optimized perioperative blood conservation strategies to further refine blood management protocols for pediatric scoliosis surgery.

## Data Availability

The raw data supporting the conclusions of this article will be made available by the authors, without undue reservation.

## Glossary

AIC: Akaike information criterion
ALT: alanine aminotransferase
APTT: activated partial thromboplastin time
ASA: American Society of Anesthesiologists
AST: aspartate aminotransferase
AUC: area under the receiver operating characteristic curve
BUN: blood urea nitrogen
CI: confidence interval
Hb: hemoglobin
HL: Hosmer–Lemeshow
IQR: interquartile range
INR: international normalized ratio
LASSO: least absolute shrinkage and selection operator
MAR: missing at random
OR: odds ratio
PMM: predictive mean matching
PSF: posterior spinal fusion
RBC: red blood cell
RCS: restricted cubic splines
ROC: receiver operating characteristic
TRIPOD: transparent reporting of a multivariable prediction model for individual prognosis or diagnosis.
