# Supplementary material for: Development of a preoperative risk score for predicting blood transfusion in pediatric scoliosis surgery: a two-center retrospective cohort study
Source: Front Med (Lausanne). 2026 Apr 23;13:1838600. doi: 10.3389/fmed.2026.1838600 (PMC13149296; doi:10.3389/fmed.2026.1838600)
Supplement: Supplementary file 1 [file Supplementary_file_1.docx]

**Supplementary Table 1: Performance comparison of predictive models for RBC transfusion in training and validation sets**

| Metric | Model 1 | | Model 2 | | Model 3 | |
| --- | --- | --- | --- | --- | --- | --- |
|  | Training Set | Validation Set | Training Set | Validation Set | Training Set | Validation Set |
| Discrimination (AUC) | 0.83 ± 0.004  (95%CI: 0.806-0.854) | 0.813 | 0.825 ± 0.004  (95%CI: 0.801-0.849) | 0.811 | 0.821 ± 0.004  (95%CI: 0.797-0.846) | 0.813 |
| Sensitivity | 0.745 ± 0.019 | 0.804 | 0.747 ± 0.016 | 0.769 | 0.79 ± 0.03 | 0.735 |
| Specificity | 0.792 ± 0.023 | 0.688 | 0.780 ± 0.018 | 0.677 | 0.73 ± 0.03 | 0.74 |
| *P*-value for HL test | 0.401 ± 0.269 | 0.277 | 0.573 ± 0.289 | 0.171 | 0.516 ± 0.308 | 0.124 |

Model 1: Multivariable logistic regression model constructed via univariable pre-screening followed by variable selection using the Akaike Information Criterion (AIC) to retain significant predictors.

Model 2: Multivariable logistic regression model built using variables selected by LASSO regression (Least Absolute Shrinkage and Selection Operator) at the lambda value corresponding to one standard error above the minimum cross-validation error (lambda 1se).

Model 3: Simplified multivariable logistic regression model derived from Model 2. Three variables were further excluded based on AIC and significance assessment, retaining four variables with no significant loss of performance. Intended for development of a simplified risk scoring system.

Training set metrics represent pooled estimates (mean ± standard deviation) across 10 imputed datasets. Validation set metrics are from a single independent cohort with complete-case analysis (samples with missing values excluded). RBC, red blood cell; AUC, area under the receiver operating characteristic curve; HL, Hosmer-Lemeshow.

**Supplementary Table 2: Transfusion rates by total risk score.**

| **Total Score** | **Training Set** | | **Validation Set** | |
| --- | --- | --- | --- | --- |
|  | Number of patients | Transfusion rate (%) | Number of patients | Transfusion rate (%) |
| 0 | 513 | 5.65 | 19 | 5.26 |
| 1 | 1183 | 3.38 | 33 | 0 |
| 2 | 13 | 76.90 | 0 | N/A |
| 3 | 1268 | 8.20 | 29 | 13.80 |
| 4 | 2880 | 13.10 | 84 | 8.33 |
| 5 | 719 | 23.40 | 15 | 20.0 |
| 6 | 923 | 21.90 | 22 | 40.90 |
| 7 | 961 | 36.10 | 32 | 21.90 |
| 8 | 1475 | 46.20 | 42 | 31.0 |
| 9 | 667 | 51.60 | 11 | 36.40 |
| 10 | 765 | 41.20 | 15 | 53.30 |
| 11 | 1206 | 68.90 | 20 | 70.0 |
| 12 | 275 | 74.90 | 6 | 50.0 |
| 13 | 522 | 75.70 | 10 | 80.0 |
| 14 | 110 | 90.90 | 10 | 70.0 |
| 15 | 150 | 86.70 | 5 | 100 |
| 16 | 100 | 90.0 | 1 | 100 |
| 17 | 120 | 100 | 1 | 100 |
| 18 | 80 | 100 | 1 | 100 |
| 19 | 0 | 0 | 0 | N/A |
| 20 | 20 | 100 | 0 | N/A |

Total scores range from 0 (lowest risk) to 20 (highest risk). The 'Number of patients' column indicates the frequency of each total score within the respective dataset. Transfusion rate represents the proportion of patients who received perioperative allogeneic RBC transfusion among those with the same total score. Training set data are pooled results from 10 multiple imputation datasets. Validation set data are derived from a complete-case analysis, where all cases with any missing data were excluded.
